# A Micromachined Coupled-Cantilever for Piezoelectric Energy Harvesters

**DOI:** 10.3390/mi9050252

**Published:** 2018-05-21

**Authors:** Agin Vyas, Henrik Staaf, Cristina Rusu, Thorbjörn Ebefors, Jessica Liljeholm, Anderson D. Smith, Per Lundgren, Peter Enoksson

**Affiliations:** 1 Department of Microtechnology and Nanoscience (MC2), Chalmers University of Technology, Kemivagen 9, 41258 Gothenburg, Sweden; v96staaf@chalmers.se (H.S.); smdavid@chalmers.se (A.D.S.); per.lundgren@chalmers.se (P.L.); peter.enoksson@chalmers.se (P.E.); 2 Ri.Se Acreo AB, Arvid Hedvalls Backe 4, 41133 Gothenburg, Sweden; cristina.rusu@ri.se; 3 MyVox AB, Isafjordsgatan 22 (c/o Ri.SE), SE-164 40 Kista, Sweden; Thorbjorn.Ebefors@myvoxultrasonics.com; 4 Silex Microsystems AB, Bruttovagen 1, 175 43 Jarfalla, Sweden; jessica.liljeholm@silex.se

**Keywords:** piezoelectric micro-energy harvester, lead zirconate titanate, bandwidth broadening, coupled cantilevers, enhanced stress distribution, finite element modeling, microelectromechanical systems (MEMS)

## Abstract

This paper presents a demonstration of the feasibility of fabricating micro-cantilever harvesters with extended stress distribution and enhanced bandwidth by exploiting an M-shaped two-degrees-of-freedom design. The measured mechanical response of the fabricated device displays the predicted dual resonance peak behavior with the fundamental peak at the intended frequency. This design has the features of high energy conversion efficiency in a miniaturized environment where the available vibrational energy varies in frequency. It makes such a design suitable for future large volume production of integrated self powered sensors nodes for the Internet-of-Things.

## 1. Introduction

With recent advancements in the field of micro electro mechanical systems (MEMS), sensors have been miniaturized with increasing functionality [1]. These sensors, due to their size, can easily be placed in exotic or unreachable locations, unthinkable before to convey useful information. These smart devices need power supplies to function, which are usually provided by batteries and chemical fuels [2]. However, once the fuels run out, the sensors need to be retrieved and provided with a new power supply. Since many of the sensors are often placed in remote and/or harsh environments, the task of carrying out the change in batteries becomes expensive. Having a miniaturized power supply that recharges itself on the basis of the energy present in the surroundings will give the future internet-of-things (IoT) access to several relevant and challenging locations—inside machines, constructions or in living tissue.

Miniaturiazation of devices is essential for the design and fabrication for IoT applications. Micro-machining fabrication techniques have been used in manufacturing complementary metal-oxide-semiconductor (CMOS) devices, sensors, actuators, and also energy harvesters at a large scale. The simplest design for micro-energy harvesters is a single cantilever with a thick proof mass hanging at the free end. Table 1 refers to several single cantilever designs explored in literature along with their dimensions, resonant frequencies and voltage and power outputs. The problem with these single cantilever based energy harvesters is the absence of active piezoelectric area along the free ends of the beam. Another serious drawback in single cantilever designs is their small bandwidth. Their performance depends on matching the resonance frequency with the vibration frequency of mechanical excitation of the object or surrounding environment whose energy is to be harvested. Even a slight shift from resonance reduces the power output drastically. There is a need for broader bandwidth devices in order to account for the random shifts of vibrational frequency. Thus, solutions that increase the range of working frequencies of a device and which can subsequently be assimilated in a MEMS structure are required.

Among potential solutions to the bandwidth and power problem are nonlinear generators with bistable structures. Stanton et al. [13] incorporated a magnetic nonlinearity with the help of two magnets strategically placed in a linear system. Wu et al. [14] and Erturk et al. [15] arrived at a similar solution of obtaining a broad bandwidth. Mann et al. [16] investigated nonlinear systems that utilized magnetic levitation to produce an oscillator with tunable resonant frequencies. Mahmoudi et al. [17] proposed a double clamped cantilever beam with a movable magnetic proof mass which is excited by oppositely polarized magnetic fields at the top and bottom. Furthermore, Yang et al. [18] designed a triple proof mass-magnet system to develop a multifrequency generator. The magnetic fields from the copper coils acted on the proof masses induced different modes of vibration in the cantilever beam which led to three close eigenfrequencies and improved bandwidth. Similarly, Sari et al. [19] demonstrated a microcantilever array consisting of 35 cantilevers of varying resonant frequencies acting in presence of a magnetic field. The relative motion between a magnet and coils fabricated over the cantilevers generated power, while the array provided the wideband harvesting frequencies. Abed et al. [20,21] expounded on the multimodal equilibrium techniques by theoretically analyzing doubly clamped cantilevers coupled with a magnetic array to develop a non-linear magnetic stiffness in the beams. Although the above described techniques could provide enhanced bandwidths, the magnets are usually large and require an auxiliary support structure that cannot be easily placed in a MEMS design. Similarly, Blystad [22] and Liu [23] suggested amplitude limiters, where a mechanical stopper is used to limit the displacement of the cantilevers. Soliman et al. [24] experimented on such a structure with a single stopper and showed a 240% improvement in the power output bandwidth of the device. Although these devices offer improved bandwidth, in practicality, a lower maximum output power and fatigue-induced failures in such designs make their incorporation harder in microstructured harvesters.

An effective approach to bandwidth broadening is using two-degree-of-freedom (2DOF) structures and reducing the gap between the first two natural frequencies. To devise natural frequencies the are closer together, Jang et al. [25] (2011) developed a 2DOF piezoelectric energy harvesting device which exploited the structure’s translation and rotation vibration modes. The device showed two-peak power output and displayed 31.8% bandwidth improvement at the power level of 155.6 mW compared to the conventional single-degree-of-freedom (1DOF) device. Kim et al. [26] (2011) demonstrated the performance comparison between a 2DOF and conventional 1DOF device at 10.9 Hz, with the the former exhibiting a 280% increase in bandwidth at a voltage level of 55 V/g. Wu et al. [27] proposed a “cut-out” 2DOF harvester with a secondary beam enclosed within the main beam, which achieves two close resonances with significantly large amplitudes. Improvement of bandwidth through 2DOF structures can be enhanced by increasing the distribution of stress on these cantilever beams. Studies by Staaf et al. [28,29] provide an assessment of using parallel cantilevers coupled to one another at one end. This improves the stress distribution patterns and the bandwidth through coupled resonance at frequencies near the natural excitation. Thus, using a 2DOF structure with a highly distributed stress can support a larger bandwidth of harvestable frequencies.

In recent years, the focus of the vibrational microenergy harvester research has shifted to fabrication of specialized designs to improve the bandwidth of the energy providing frequencies. Park et al. [9] designed an intrinsically stress-induced bent silicon cantilever to study on the principle of proportional dependence of the output power on the bending moment. They also shaped the cantilevers in a trapezoidal form to improve the distribution of stress on the beam. Leuke et al. [10] fabricated a set of folded spring structures for reduction of the operational frequency of the microstructures. The folded beam shape reduces the overall stiffness of the design and thus bring the natural frequency of the system down to 30–300 Hz. Yu et al. [11] designed a five cantilever system with a single large proof mass. The fabricated generator had plates and a silicon proof mass. A similar concept was also employed by Zhang et al. [12] where, instead of rectangular folded springs, they used circular annular rings, each attached to the central proof mass.

**Figure 1:**
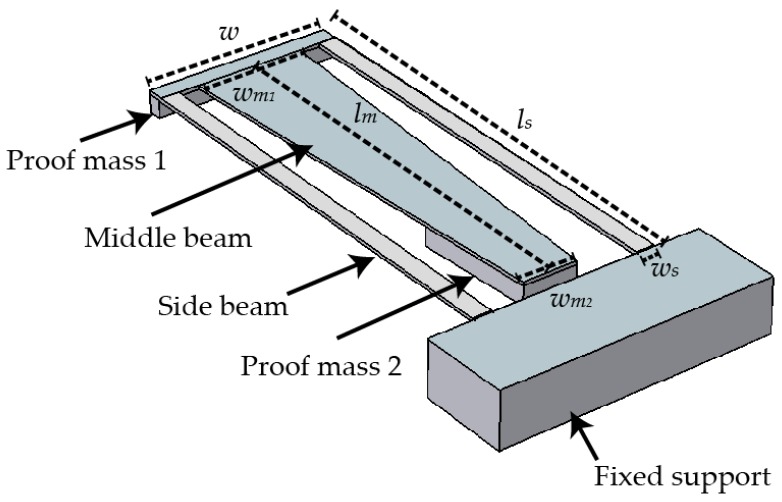
Schematic image of the *M-shape* harvester design. Table 2 outlines the dimensions of the design.

Based on the review of techniques, it is evident that usage of a 2DOF structure for bandwidth improvement with coupled cantilevers designed in a form that enhances stress distribution is desirable. The aim of this study was to complement and translate these macro-concepts into a micro-energy harvester. An *M-shaped* micro-design was analyzed, simulated, and fabricated (Figure 1). It incorporates the advantages of a 2DOF design in a trapezoidal shaped middle beam coupled with rectangular cantilevers in a single structure. The *M-shape* is optimized to achieve a compact structure with a uniquely uniform stress distribution across the beams and a higher bandwidth of utilizable frequencies through reducing the gap between the first two natural frequencies. Fabrication of the coupled resonators with a lead zirconate titanate (PZT) layer was performed and assessed. PZT was chosen as the piezoelectric material as it has more than ten times higher figure-of-merit than AlN or ZnO [30]. The feasibility of using micromachining to realize the *M-shape* design has been demonstrated by continuous evaluation of the progress during the fabrication process and by matching the observed mechanical behavior of the device to numerical simulations and to a simple analytical model.

## 2. Theoretical Design and Simulation

### 2.1. Theory

**Figure 2:**
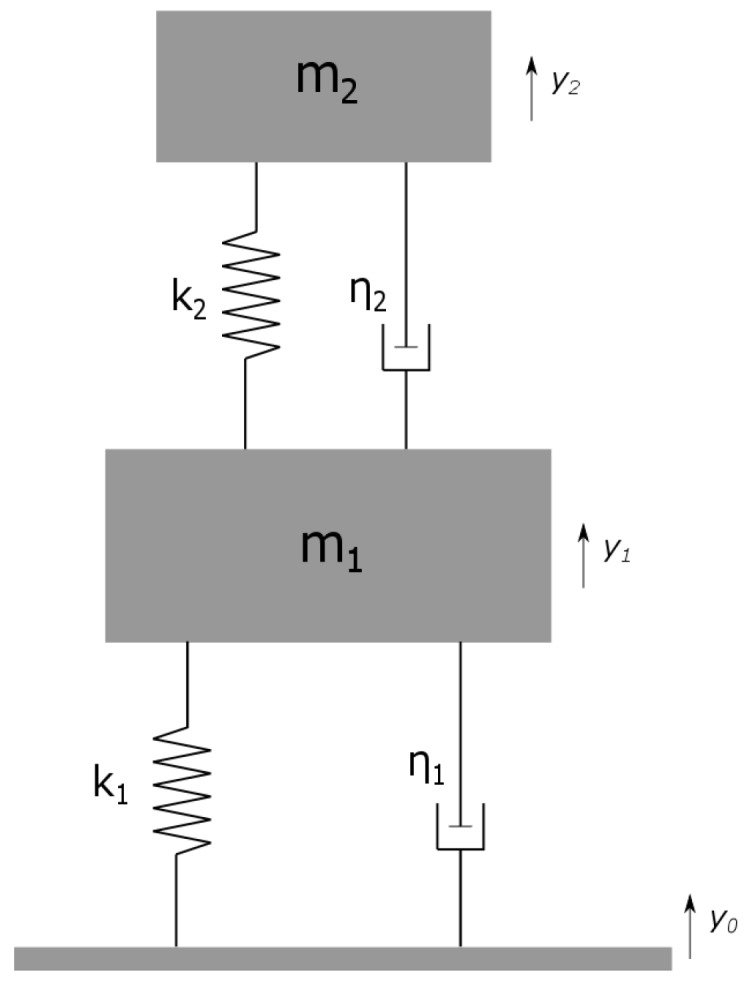
Typical lumped equivalent mass–spring–damper model for two-degree-of-freedom (2DOF) cantilever design.

**Figure 3:**
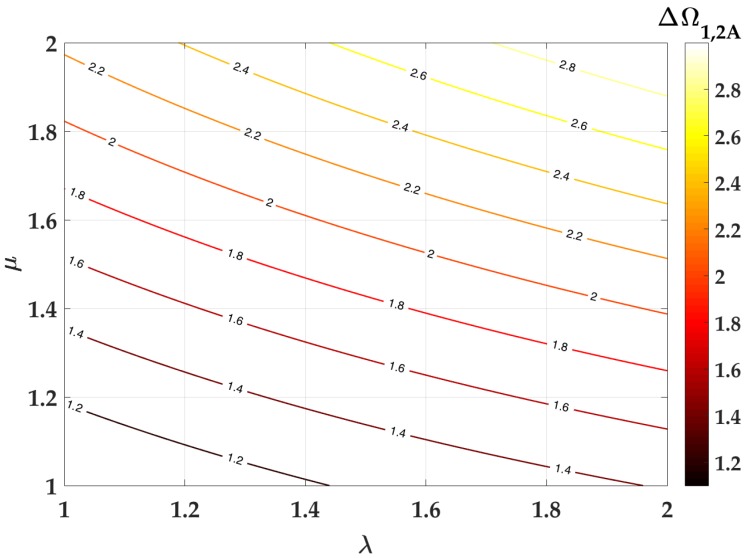
Contour plot of difference in first two dimensionless eigenfrequencies ($\Delta \Omega_{1,2A}$) with respect to ratio of beam masses (μ) and their respective first eigenfrequencies (λ) acquired from Equation (1).

Piezoelectric energy harvester cantilevers are normally modeled as a spring–mass–damper system. The harvester design in Figure 1 can be translated into the schematic in Figure 2a, which is a conventional 2DOF lumped parameter model used for the analysis of the *M-shape* designs. In this model, $m_{1}$ and $m_{2}$ are masses of the primary side beams and secondary middle beam structures, respectively; $k_{1}$, $k_{2}$, and $\eta_{1}$, $\eta_{2}$ are their respective spring constants and dampings. When the system is in a base excited configuration, the initial displacements of the base, primary, and secondary proof masses are $y_{0}$, $y_{1}$ and $y_{2}$, respectively. The model is elaborated further by Tang et al. [31]. On solving the equations of motion for negligible damping, the dimensionless difference in eigenfrequencies ($\Delta \Omega_{1,2A}$) is calculated as:

(1) $$\Omega_{1,2A}=\sqrt{\frac{\left(1+\mu \right)\lambda^{2}+1}{2}\pm \frac{\sqrt{{\left(\left(1+\mu \right)\lambda^{2}+1\right)}^{2}-4\lambda^{2}}}{2}}$$

where μ = $\frac{m_{2}}{m_{1}}$ and λ = $\frac{\omega_{2}}{\omega_{1}}$. $\omega_{1}$ and $\omega_{2}$ are the natural frequencies of the two systems vibrating separately which are calculated as $\omega_{1,2}$ = $\sqrt{\frac{k_{1,2}}{m_{1,2}}}$. It is evident that the closeness of the first two natural frequencies depends on the parameters μ and λ. The minimum values for $\Delta \Omega_{1,2A}$ are demonstrated for μ, λ< 1. However, considering the fabrication restraints in designing a 2DOF miniaturized cantilever, λ will typically be greater than 1, as the middle beam is always shorter than the primary beams. Therefore, for COMSOL analysis, the dimensions are considered in a way that they lie within the window 1 < λ < 2. Fabrication design considerations for a distributed stress structure also limit the values of μ> 1 as the volume of the middle beam is higher. Figure 3 shows the region in the contour plot of $\Delta \Omega_{1,2A}$ vs. μ and λ from Equation (1). The trend given from this simple analytical analysis is that values of μ and λ approaching 1 would yield a small separation of the coupled structures resonance peaks. According to Tang [31], the damping ($\eta_{1}$) in the primary part is more influential in the outcome amplitudes at the resonant frequencies. The effect of damping will need to be taken into account when evaluating the harvesting efficiency once we start extracting electrical power from the system. The next section describes the simulation results in COMSOL (version 5.2, COMSOL, Inc., Stockholm, Sweden) based on this window of operation for μ and λ.

### 2.2. Cantilever Design

The 2DOF microenergy harvester was numerically simulated in COMSOL to acquire the optimal dimensions for the realization of enhanced stress distribution and to make the bandwidth broader. Several different topologies were investigated. However, keeping manufacturability as a core issue, a single design named *M-shape* was explored in more detail. Figure 1 shows the schematic of the generalized harvester in COMSOL. The big block was taken as the fixed support in the simulation. This is used as a simulation tool for the support structure that was included in fabrication to improve the robustness of the design. To complete a MEMS piezoelectric energy harvester, the design structure consists of silicon combined with piezoelectric material which is chosen to be PZT-5A. The five main dimensional parameters that play an important role in determining natural frequencies are the length and width of the side beams, $l_{s}$ and *w*, respectively; the length of middle beam, $l_{m}$; and the widths of the middle beam at the attached and free ends, $w_{m1}$ and $w_{m2}$, respectively. The thickness of the device is decided by the device layer in the silicon on insulator (SOI) wafer used, i.e., 20 μm. The thickness of the proof mass was 100 μm for each design so that they could be fabricated in a single process plan.

With the proof mass thickness 100 μm as a fabrication design parameter, the dimensions $l_{s}$ and *w* govern the natural frequency of the system. They were chosen as $l_{s}$ = 2900 μm and *w* = 100 μm such that the device resonance frequency is in the range 1.2–1.5 kHz and it has enough area for the middle beam compartmentalization. The simulation gives $\omega_{1}$ = 1619 Hz for the generated primary structure (side beams with proof mass).

**Figure 4:**
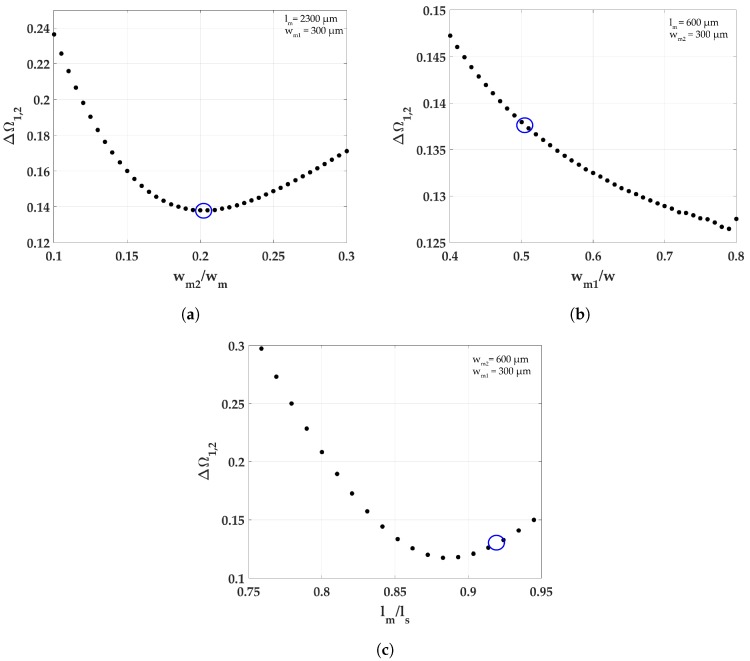
Simulated variation of $\Delta \Omega_{1,2}$ with respect to: (**a**) $w_{m2}$, width of the free end; (**b**) $w_{m1}$, width of fixed end; and (**c**) $l_{m}$, length of the middle beam. The blue circle denotes the values of *M-shape* on the respective graphs.

The μ values were obtained using $m_{1}$ and $m_{2}$, the masses of the side and middle cantilevers respectively. The masses were calculated as ρV, where ρ is the density of the material (silicon) and *V* is the volume from the structure shown in Figure 1. This forms the basis for the dimensions $l_{m}$ = 2300, $w_{m2}$ = 300, and $w_{m1}$ = 600. Figure 4 shows the effect of $l_{m}$/$l_{s}$, $w_{m1}$/*w* and $w_{m2}$/*w* on $\Delta \Omega_{1,2}$, where $\Delta \Omega_{1,2}$ = $\frac{\Delta f_{1,2}}{\omega_{1}}$. In Figure 4b,c, the datapoints for $l_{m}$ and $w_{m2}$ in the region $\Delta \Omega_{1,2}<$ 0.16 are in the range 2440–2700 μm and 160–260 μm, respectively. The value for $w_{m1}$ was chosen to reduce the stress between the side beams and the trapezoidal middle beam. The chosen dimensions are shown in Table 2. The simulation for $\Delta \Omega_{1,2}$ shown in Figure 4 suggests a significant difference in the order of magnitude with the acquired values in Figure 3, since the values calculated for μ and λ in the design are μ = 1.22 and λ = 1.48, which gives a simulated $\Delta \Omega_{1,2}$ of 0.14, and an analytical $\Delta \Omega_{1,2A}$ of 1.52. Despite this fact, we see the same trend for optimizing λ and μ from the simulations. Therefore, although the representation of the actual structure into a lumped 2DOF model would require more elaboration to be faithful, the simple model presents a useful starting point for design considerations.

### 2.3. Simulation Results

**Figure 5:**
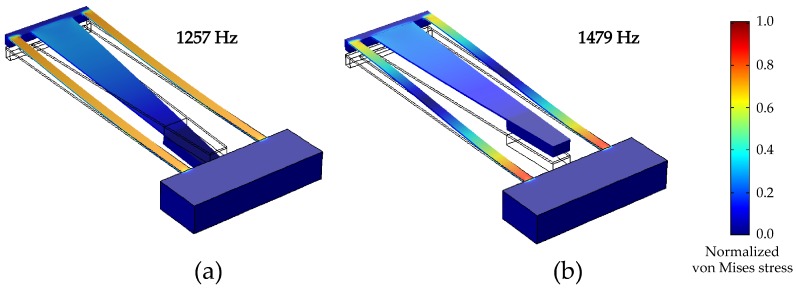
Mode shapes of (**a**,**b**) *M-shape* at the first and second natural frequencies. The gradient on the side displays the normalized von Mises stress on the cantilever beams without the lead zirconate titanate (PZT) piezoelectric layer.

**Figure 6:**
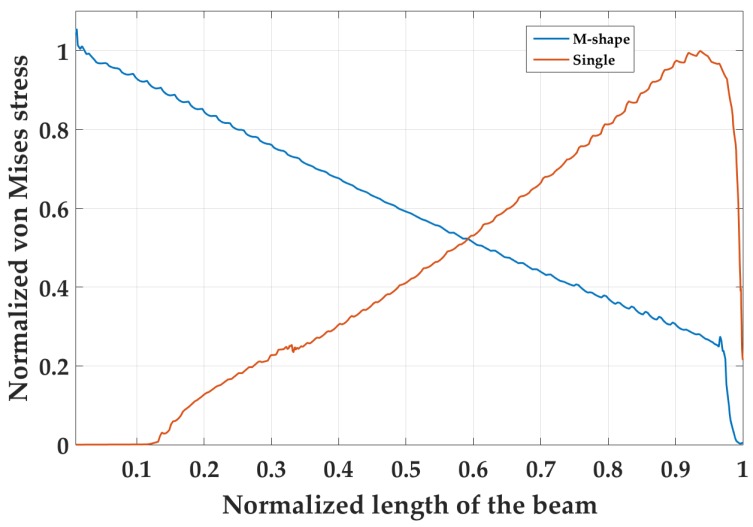
Stress distribution on *M-shape* across the side beam length compared to a single cantilever at the same natural frequency of 1257 Hz.

The results for the eigenfrequency simulations are shown in Figure 5. For the *M-shape*, the first and second eigenfrequencies are observed at 1257 Hz and 1479 Hz, i.e., $\Delta \Omega_{1,2}$ = 0.14. The normalized stress gradient suggests that the vibration of the middle beam enhances the distribution of stress on each of the two side beams. The middle beam does not act as a dormant proof mass; it has its own characteristic vibrational mode. The presence of stress on the middle beam is coupled with the side beam’s stress, which leads to a larger area acting under stress. Figure 6 displays the distribution of stress on the outer edge of the cantilever side beams compared to a single cantilever at the same resonance frequency of 1257 Hz. The free end of the beam is at x=0. The boundary conditions for each design were kept constant. The single cantilever has the maximum stress at its fixed end. There is negligible stress on the beam at its free end. In contrast, the *M-shape* displays a different characteristic curve where the stress is significant over its whole length.

## 3. Fabrication

The process plan used in our fabrication is explained in two main parts: layering and patterning of the electrodes and piezoelectric layers, and etching of the silicon substrate to create the cantilever design. The electrodes and PZT were prepared by SILEX Microsystems (Jarfalla, Sweden) on an 6” silicon-on-insulator (SOI) wafer.

### 3.1. Process

Fabrication began with a 500 μm silicon-on-insulator (SOI) wafer. A layer of thin 500 nm SiO${}_{2}$ was thermally grown onto the SOI wafer. The SiO${}_{2}$ layer was then patterned using a standard photolithographic process. The uncovered SiO${}_{2}$ was etched away with a mixture of CHF${}_{3}$ and O${}_{2}$. A 20 nm layer of titanium and a 100 nm layer of platinum were sputtered to create the bottom electrode. A buffer layer of LaNiO 20 nm was deposited on top of the bottom electrodes. Then, a lead zirconate titanate (PZT) layer of 1.1 μm was deposited by a sol-gel process on the buffer layer.

**Figure 7:**
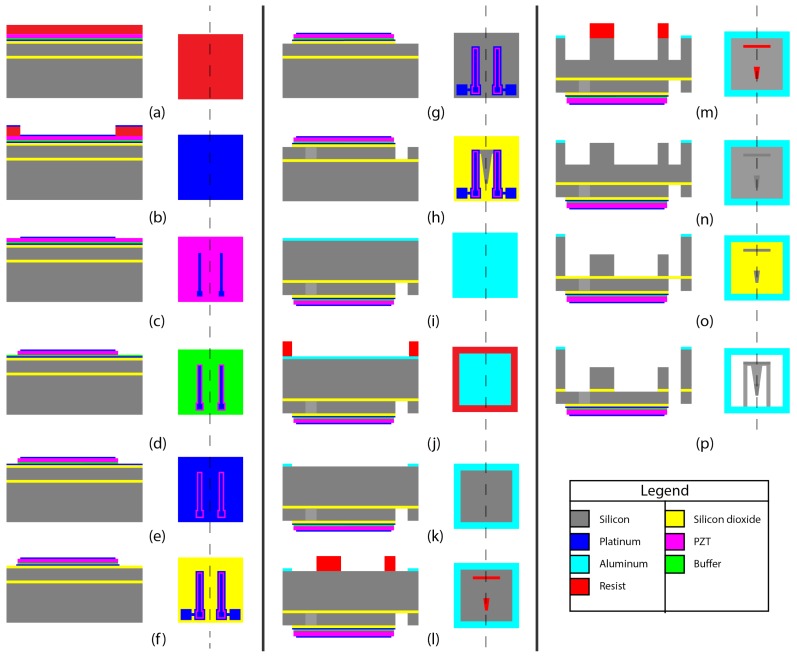
Schematic process plan for fabricating cantilevers. The left side on each panel is a cross section of the top view of the substrate. *Topside Processing*: A 6” SOI wafer with 1.1 μm lead zirconate titanate (PZT), 100 nm buffer oxide and 120 nm bottom electrodes: (**a**) photoresist spinning; (**b**) Pt/Ti deposition on patterned resist; (**c**) top electrode lift-off; (**d**) PZT wet etching; (**e**) buffer oxide etching; (**f**) bottom electrode etching; (**g**) photoresist spinning to protect the electrodes; (**g**) SiO${}_{2}$ etching; and (**h**) silicon DRIE etching, 20 μm. *Backside processing*: (**i**) Aluminum sputtering; (**j**) resist deposition for aluminum etching; (**k**) aluminum etching for the second hard mask; (**l**) photoresist hard mask for proof mass protecting during etching; (**m**) silicon deep reactive-ion etching (DRIE), 100 μm; (**n**) photoresist mask removal; (**o**) silicon DRIE, 280 μm; and (**p**) SiO${}_{2}$ etching and realizing the microcantilevers as in Figure 1.

The electrode stack was patterned, one layer at a time. The wafer was initially preheated and MicroChem ma-N1410 resist (MicroChem, Westborough, MA, USA) was spin coated with post softbake, exposure and development in ma-D33 (Figure 7a). Thin films of platinum and titanium of 100 nm and 20 nm, respectively, were deposited on the wafer surface through evaporation (Figure 7b). The resist was lifted off in mr-REM 400 bath in ultrasonic environment for 1 h (Figure 7c). The 1.1 μm PZT and 100 nm buffer were wet etched in a 1:1:20 solution of hydrogen fluoride (HF)(7%):HNO${}_{3}$(6%):H${}_{2}$O [32] (Figure 7d,e). The bottom electrodes had to be etched at a high 400 W radio frequency (RF) power in 10 mTorr pressure at 25 sccm argon flow rate. Thermally grown SiO${}_{2}$ of 500 nm below the electrodes was etched along in the same process. The wafer was processed in the load lock chamber for 25 min in total, 5 min for Ti etching, and 20 min for SiO${}_{2}$ (Figure 7f,g).

The cantilever structures were created by etching 20 μm of silicon from the front side, and 100 μm and 280 μm etching from the backside. To create the cantilevers in the 20 μm device layer of the SOI wafer, AZ4562 thick resist (MicroChemicals GmbH, Ulm, Germany) mask was developed. The exposed surface was etched to 21.6 μm in Centura II (DPS and MxP, HD Pacific, Inc., Mukilteo, WA, USA) (recipe in Table A2) deep reactive ion etching tool (Figure 7h). The wafer was then treated from the backside to realize the proof masses and release the structure. A hard mask of aluminum was created to make the support frame. A 0.5 μm aluminum layer was sputtered and etched out through a resist mask aligned to the top side using the backside alignment technique (Figure 7i). Aluminum was etched using H${}_{3}$PO${}_{4}$ at 40 °C in 3 min and 30 sec(Figure 7k). The initial thermally grown SiO${}_{2}$ layer was then etched using CHF${}_{3}$/Ar at 250 W. A thick resist mask of AZ4562 was patterned for the proof masses. With the resist mask, the exposed backside silicon was etched in STS-ICP DRIE (MechSE-Illinois, Urbana, IL, USA) chamber to 100 μm (Figure 7l). The etch recipes are shown in Table A1 in Appendix A. Resist was stripped in mr-REM400 under ultrasonication (Figure 7m). A thermal tape was used to attach the substrate to a carrier wafer. The remaining 280 μm of handler SOI wafer was etched (Figure 7n). The wafer was then mechanically cleaved into small chips each containing one micromachined design (Figure 7o).

### 3.2. Challenges in Fabrication

This section describes the experiments and challenges experienced in the microfabrication of the *M-shape* energy harvesters.

**Figure 8:**
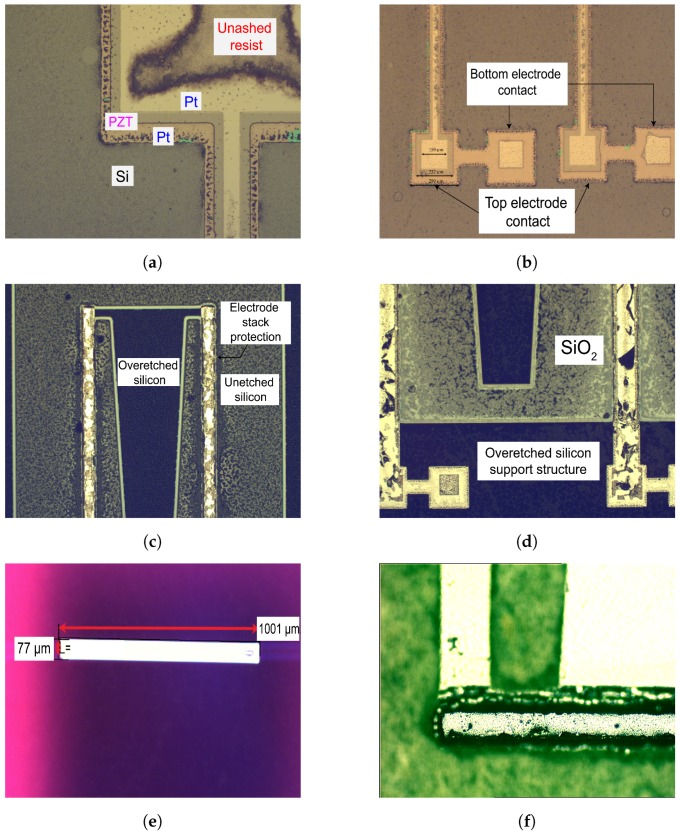
(**a**) Electrode layout after the top electrode fabrication process steps under an optical microscope for a single cantilever; (**b**) Contact pads for each side beam of *M-shape* cantilevers. The intention is to connect the two electrodes in series; (**c**) Presence of unetched silicon due to uneven etching of the wafer; (**d**) Areas where SiO${}_{2}$ is visible after topside etching; (**e**) Proof mass on *M-shape* realized after the first backside etch process; (**f**) Etched proof mass connected to topside cantilever structure after wafer-through etch.

The first challenge was the fabrication of the electrodes. Platinum, being an unreactive metal, cannot be easily removed from the substrate through conventional reactive ion etching recipes. The top electrode was deposited with ease through a lift-off process. However, for the bottom electrode, dry etching was the only alternative. Wet etching of bottom electrodes is ill-advised as it could potentially etch the PZT through the sides and lift the entire stack off. Therefore, a 400 W high-energy argon etching recipe was created in PlasmaTherm RIE that had an etch rate of 11 nm/min. Figure 8a shows the electrode stack created after lift-off of top electrodes, wet PZT etching, and dry etching of bottom electrodes. The dark blackish-grey region in the image is the unashed resist from the resist mask used. Figure 8b displays the bond-pads after electrode fabrication. The left side bond-pads are for the side beam on left, and likewise for the right side contact pads. Spacings of 35 μm and 30 μm were created between the top electrode and the PZT pattern, and PZT and bottom electrode pattern respectively. The argon 400 W recipe is an efficient recipe to remove nearly any sort of thin film. During the bottom electrode etching, the wafer was subjected to the machine for 35 min. During this time, the recipe went through 100 nm platinum, 20 nm of titanium and 400 nm of SiO${}_{2}$ without affecting the resist in any considerable way. The only observable change in the photoresist was its color and transparency.

The second challenge in the fabrication was observed in the topside silicon etching. This was due to the uneven heating of the substrate during the deep reactive-ion etching (DRIE) process in Centura II. Centura II machine parameters can be found in Table 2. Typically, in a DRIE process, the wafer is cooled at the carrier to maintain a constant etch rate. The backside of the substrate was mounted on an 8” wafer through a thermal tape. The formation of bubbles between the tape and substrate interface led to an uneven cooling of the wafer, which resulted in evaporation of photoresist at the heated surfaces before the whole etching was completed. This led to a deviation in the thickness of the beams. The areas which were protected by the electrode pads were etched until the buried SiO${}_{2}$ layer, while the areas exposed after resist evaporation had a depth of around 8 μm to 16 μm depending on the area’s position on the 6” wafer. In Figure 8, images taken under an optical microscope demonstrate the extent of destructive processing carried out during DRIE silicon etching. There are areas on the wafer which are underetched (Figure 8c) and some areas where the buried oxide layer is visible (Figure 8d). Although the non-uniform etching severely affected the yield on the wafer, in the end, the wafer could still be processed further on to create the coupled cantilever structure. The dimensions accrued from optical characterization were as follows: $l_{s}$ = 2908 μm; *w* = 1001 μm; $l_{m}$ = 2746 μm; $w_{m1}$ = 481 μm; $w_{m2}$ = 98 μm.

The final challenge encountered during the wafer processing was the backside etching. The details for the STS machine parameters can be found in Table 2. This process step, as explained previously, involved two DRIE etching steps, one to create the proof masses of 100 μm thickness and another to etch through the full wafer thickness, i.e., 280 μm. The proof masses were etched, and their optical image is shown in Figure 8e. Their thickness was measured in a Dektak Profiler (Bruker Corporation, Billerica, MA, USA). During the final etching process, the exposed silicon surface was etched taking the proof mass design downwards (Figure 8f. The most significant challenge experienced in this process was the uneven etching of the outer edges of the wafer compared to the inner. Their etch rates were 4.1 μm/min and 3.25 μm/min, respectively. Thus, the outer proof masses differed up to 21% in thickness from the inner ones. Furthermore, after the backside etch, the wafer system comprising of the main wafer taped to the carrier wafer is heated to 120 °C which led to a difficulty in releasing the cantilevers.

**Figure 9:**
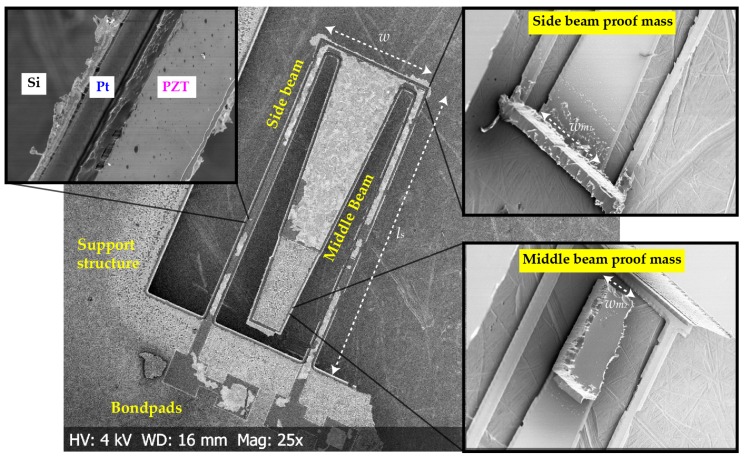
Scanning electron microscope (SEM) image of *M-shape* at 25× magnification. Insets contain the backside processed images of the primary and secondary proof masses, and the PZT layer on the bottom electrodes on the side-beam cantilevers.

An scanning electron microscope (SEM) image of *M-shape* is shown in Figure 9. The shiny surface on top is the top electrode of platinum which is not covering the PZT surface completely, especially on the side beams. A step-like structure was created during the topside etching, which is visible at the interface between the electrode stack and silicon on the middle beam. Insets on the right side show the proof masses at the bottom. The top surface and intended structure of proof masses were well preserved. However, the side profile shows the troughs created in silicon through DRIE etching. The notching effect at the side beams, visible in the insets, are formed by over-etching of silicon through the etchant gases. With no end-point detector for SiO${}_{2}$ in the machine, the charged ions deflect at the insulator interface towards the silicon surface leading to a sideways etching. This phenomenon has been described by Laermer et al. [33]. The SEM images demonstrate that the design can be fabricated with the above-described processing steps. However, they require optimization for a better yield.

## 4. Results and Discussion

### 4.1. Mechanical Characterization

The fabricated designs were measured for mechanical characterization in a laser doppler vibrometer (LDV) setup. An LDV uses monochromatic light to measure the velocity of a moving object by scattering light off the surface of the object and measuring the frequency shift in the reflected light. This detection is made interferometrically and leads to very accurate velocity measurements. The motion is excited by a shaker which is driven by the LDV internal signal generator and an amplifier. The fact that the shaker excitation is phase locked to the detection implies that phase sensitive measurements can be carried out, showing how different parts of the structure moves relative to each other.

**Figure 10:**
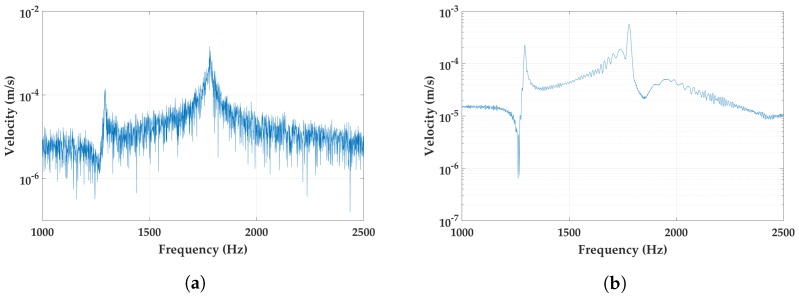
Characterization of *M-shape* in a laser doppler vibrometer (LDV) setup in different excitation signals: (**a**) Gaussian white noise of 0.1 V amplitude; and (**b**) periodic chirp voltage of 0.1 V. Both measurements show the same resonance frequencies.

Figure 10 shows the velocity response in the measured frequency domain for the free end of the *M-shape* device. In the first graph (Figure 10a), the response is recorded under a Gaussian white noise excitation across a frequency spectrum of 3.5 kHz. The first eigenfrequency of the design is measured at 1294 Hz, which is very close to the result of the simulation. The second peak, however, resides higher in frequency than predicted at 1781 Hz. Figure 10b shows the device when it is subjected to a periodic chirp of excitation across 3.5 kHz.

### 4.2. Discussions

The fabricated structure’s first eigenfrequency is well predicted by simulations. However, the second peak displayed in simulations at 1479 Hz has the actual second eigenfrequency at 1781 Hz. The fabricated device thus has a higher $\Delta \Omega_{1,2}$ = 0.3 compared to the simulated $\Delta \Omega_{1,2}$ = 0.14. The trend in Figure 3 suggests that a significant increase in λ, i.e., the ratio of the two separate eigenfrequencies $\frac{\omega_{2}}{\omega_{1}}$, causes the gap between the natural frequencies of the device to widen. An increase in λ could occur through the misalignment of proof masses with respect to the cantilever structure. When the middle proof mass shifts towards the coupling end of the cantilevers, the stiffness $k_{2}$ increases, leading to a higher $\omega_{2}$ and a higher λ.

Table 2 compares the values of the simulated and fabricated device. Applications of the proposed energy harvester can be envisaged in gas turbines and conventional machine tools. Such sources exhibit vibrations over 1 kHz while being operational. Thus, mounting the energy harvester on such sources can power wireless sensors for information gathering and condition monitoring.

## 5. Conclusions

An M-shaped 2DOF cantilever for energy harvesting was designed based on the principle of closing the gaps between the first two natural frequencies to achieve a broad bandwidth and improved stress distribution. The design was fabricated using micromachining techniques and was investigated for dimensional and mechanical characteristics. Dimensional analysis showed the feasibility of the fabrication process. Mechanical evaluation further demonstrated that the device behavior is close to what was intended in the design. In addition, the demonstrated *M-shape* micro-cantilever design shows harvesting capabilities in beam vibrations ranging from 1293 Hz to 1781 Hz, which can be attributed to the coupling mechanism in a single structure.

Future work can be directed towards two main features: (1) creating designs in the kHz range with minimal $\Delta \Omega_{1,2}$ based on the influence of masses and spring constant accrued from the design; and (2) formulating an improved process plan for better yield and design conformity for the middle beam in particular.

## Figures and Tables

**Table 1:** List of single cantilevers fabricated in the literature with their outputs.

Device	Size (mm)${}^{2}$	Thickness (μm)	Res. Freq. (Hz)	Vpp (V)	Power (μW)	Geometry
Lee et al. [3]	3 × 5	10	575	0.81	0.471	Single Cant.
Shen et al. [4]	4.8 ×0.4	36	461	0.16	2.15	Single Cant.
Muralt et al. [5]	1.2 × 0.8	5	870	1.60	1.4	Single Cant.
Lee et al. [6]	3 × 1.5	500	255	2.7	2.7	Single Cant.
Isakorn et al. [7]	1 × 2.5	15	2300	0.27	13	Single Cant.
Kim et al. [8]	3 × 1	21	243	0.3	2.15	Single Cant.
Park et al. [9]	3 × 2	18	115	-	1	Bent Cant.
Leuke et al. [10]	-	-	226	-	0.69	Circ. Spring
Yu et al. [11]	3 × 2.4	50	234	-	66.75	Multi Cant.
Zhang et al. [12]	6 × 6	-	<11	0.0075	-	Rect. Spring

**Table 2:** Characteristic features for simulated and fabricated *M-shape* device.

Dimensions	$l_{s}$	*w*	$l_{m}$	$w_{m1}$	$w_{m2}$	$\Delta f_{1,2}$	$\Delta \Omega_{1,2}$
Simulation	2900	1000	2700	500	100	222	0.14
Fabricated	2908	1001	2746	481	98	487	0.30

## Appendix A

**Table A1:** Etch recipes used in the STS-ICP plasma etcher.

STS-DRIE	SF6		C4F8		Pressure (%)	RF (W)	Platen (W)
	Flow (sccm)	Time (s)	Flow (sccm)	Time (s)			
SI_FASTO	130	12 + 1	85	7 + 0.5	55	600	13
SI_SLOWO	130	10 + 1	85	7 + 0.5	55	600	10

**Table A2:** Overview of the etch details in Centura II (DPS & MxP).

Centura II Parameters	Deposition	Breakthrough	Etch
Time (s)	1.2	1.3	1.5
Pressure (mTorr)	55	90	90
Bias Power (W)	5	100	50
Frequency (Hz)	125	125	125
Source Power (W)	1900	1900	1900
